# Metabolic syndrome burden, determinants and treatment status in an urban slum resettlement colony in Delhi, India

**DOI:** 10.1093/inthealth/ihae024

**Published:** 2024-03-22

**Authors:** Shivani Rao, Saurav Basu, Kajal Nandi, M M Singh, Heena Lalwani, Vansh Maheshwari, Amod Borle, Nandini Sharma

**Affiliations:** Department of Community Medicine, Maulana Azad Medical College, New Delhi 110002, India; Indian Institute of Public Health – Delhi, Public Health Foundation of India, Haryana 122102, India; Department of Biochemistry, Maulana Azad Medical College, New Delhi 110002, India; Department of Community Medicine, Maulana Azad Medical College, New Delhi 110002, India; Department of Community Medicine, Maulana Azad Medical College, New Delhi 110002, India; Indian Institute of Public Health – Delhi, Public Health Foundation of India, Haryana 122102, India; Department of Community Medicine, Maulana Azad Medical College, New Delhi 110002, India; Department of Community Medicine, Maulana Azad Medical College, New Delhi 110002, India

**Keywords:** abdominal obesity, diabetes, hypertension, India, metabolic syndrome, screening

## Abstract

**Background:**

Metabolic syndrome (MetS) in low-resource settings contributes to accentuated risk of cardiovascular disease, including stroke. The study objective was to estimate the prevalence, determinants and treatment status of MetS in an urban slum resettlement population in Delhi, India.

**Methods:**

This study was conducted from February to May 2023. Multiphase sampling was conducted with 1910 individuals screened for abdominal obesity (AO), with 996 detected as having AO, of which, 400 were selected by simple random sampling and further evaluated for triglycerides (TGs), high-density lipoprotein (HDL) and fasting glucose levels.

**Results:**

Among the 400 participants detected as having AO, 211 had evidence of MetS (52.75% [95% confidence interval 47.83 to 57.62]). The most prevalent combination of MetS clustering was for all five components (AO, diabetes mellitus [DM], hypertension [HTN], low HDL and high TGs; 14.69%), followed by AO, DM and HTN (12.32%). On adjusted analysis, the odds of having MetS was found to be independently associated with increasing age (≥40 y) but not sex.

**Conclusions:**

A high burden of MetS and suboptimal treatment status is prevalent in urban slum populations. Screening of individuals with AO, especially in those >40 y of age, can be an effective programmatic strategy for early diagnosis and management of MetS and its underlying components.

## Introduction

The increase in metabolic risk factors, namely elevated blood pressure (BP), overweight/obesity, hyperglycaemia and hyperlipidaemia are contributing to an emerging pandemic of non-communicable diseases (NCDs), especially in developing countries amidst an ongoing demographic, epidemiological, social, economic and nutritional transition.^1,2^ Evidence from the Global Burden of Disease study (2019) indicate no change in mortality from diabetes and obesity since 1990, with a disproportionately higher burden of mortality in low-income countries.^3^ In India, a lower-middle-income country with a population of 1.4 billion, NCDs were attributed to cause 61.8% of the total annual mortality in 2016.^4^ Furthermore, a recent pan-national survey estimated that India currently has >100 million patients with DM, a 36% prevalence of hypertension (HTN) and a 50% prevalence of dyslipidaemia, with urban areas having significantly higher prevalences compared with rural areas of the country.^5^

The presence of metabolic NCDs contributed to the elevated risk of metabolic syndrome (MetS), a condition characterized by physiological, clinical and metabolic abnormalities that increase by twofold the risk of cardiovascular disease, with a lifetime two–fourfold higher risk of stroke and a three–fourfold higher risk of myocardial infarction.^6–8^ Although there is no universally accepted definition of MetS, the widely accepted classification by the International Diabetes Federation considers MetS to be present in those individuals having a large waist circumference with two additional criteria among elevated fasting blood glucose, high BP, triglycerides (TGs) and high-density lipoprotein (HDL) cholesterol levels.^9^

The prevalence of MetS in India was estimated at 30% in a systematic review pooling results from 65 studies, although there is significant regional variation, with urban areas having a significantly higher prevalence compared with rural areas, possibly attributable to lower energy expenditure and different dietary and lifestyle configurations.^10,11^ Within urban areas, people living in slums and slum resettlement colonies, especially rural migrants, are at high risk of MetS and linked NCD risk factors considering their comparatively lower socio-economic status and adverse social determinants contributing to rapid and unhealthy lifestyle and nutritional transitions.^12,13^

The public health implications of a high burden of MetS in socio-economically vulnerable populations is disproportionately higher due to the associated multimorbidity, requiring optimal levels of medication adherence to multiple health conditions, including diabetes, HTN and dyslipidaemia, apart from adopting healthy dietary and physical activity patterns for maintaining good health.^14,15^ Medical management of MetS is further compromised in undiagnosed DM and HTN cases due to a lack of effective screening. Moreover, failure to initiate pharmacological therapy in patients at risk for MetS with uncontrolled plasma lipid levels further aggravates the risk of progression of cardiovascular diseases.^16^ People with MetS are also at high risk of medication non-adherence due to polypharmacy and higher pill load, patient factors such as forgetfulness, increased side effects and drug interactions and cost-related non-adherence due to limited availability and affordability of the required drugs, factors that are more likely in situations of poverty and deprivation.^17,18^ Urban slum populations are consequently at higher risk of the incidence and progression of MetS and associated complications such as DM and cardiovascular disease, which translate into increased morbidity, mortality and healthcare costs.

Although, multiple studies have estimated the burden and predictors of MetS in India, there is a paucity of information on the prevalence and predictors of MetS in low-resource settings such as urban slums and slum resettlement areas that constitute nearly half of the urban population in India. Also, medication adherence including treatment initiation and persistence in patients with MetS has previously not been assessed in these settings in India.

We therefore conducted this study to estimate the prevalence, determinants and treatment status of MetS in a densely populated urban slum and slum resettlement population in Delhi, India.

## Methods

### Study design and setting

This community-based cross-sectional study was part of a larger study for mass NCD screening. The study site was an urban resettlement colony and slum resettlement area in the Northeast District of Delhi having an estimated population of 50 000, a site purposively selected, as it is an updated demographic, developmental and environmental surveillance site (DDESS), also being the field practice area of a government medical college.^19^

#### Study population

We screened all adults with residence at least 6 months in the area, irrespective of their medical history for HTN and abdominal obesity (AO). Participants having AO, defined as a waist circumference ≥90 cm in males and ≥80 cm in females, were considered eligible for further screening for MetS.^20^ Data were collected for a period of 4 months from February to May 2023.

#### Study outcomes

The primary outcome was the proportion of adult participants with AO having evidence of MetS. The secondary outcome was adherence to anti-diabetes and anti-hypertensive medication in patients diagnosed with MetS.

#### Sample size and sampling strategy

**Figure 1.**
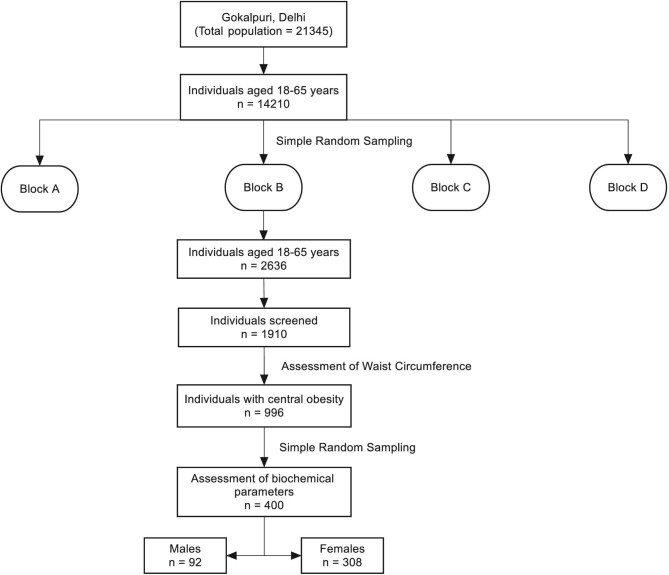
Flowchart showing the selection of adults 18–65 y of age for the present study.

The sample size calculated at 95% confidence levels, 5% absolute precision and considering 50% prevalence of MetS in the study population was 400. A multiphase sampling method was used for selection of the participants. One of the four blocks of the slum resettlement colony was selected randomly. A consecutive sampling method of the eligible participants in the block was conducted by house-to-house sampling. Among the participants detected with AO, a total of 400 were further selected using the simple random sampling method through computer-generated randomization for the purpose of biochemical testing for confirmation of MetS (Figure 1).

### Operational definitions

MetS was considered to be present in individuals with AO (defined as a waist circumference ≥90 cm in males and ≥80 cm in females) along with any two of the following parameters:^9^ TGs ≥150 mg/dl, HDL <40 mg/dl (males) or <50 mg/dl (females), fasting blood glucose ≥100 mg/dl or previously diagnosed diabetes and systolic BP ≥130 mmHg or diastolic BP ≥85 mmHg or previously diagnosed HTN.

The level of medication adherence was assessed using the previously validated four-item Morisky–Green–Levine (MGL) adherence scale. The MGL scale comprises four questions (pertaining to forgetfulness or carelessness, cessation of prescribed medications when feeling better or worse) where each item has a yes or no response. In our study, we dichotomized the full score on the MGL scale into two groups: those who scored 4 on the MGL scale were considered as adherent and those with scores <4 were considered as non-adherent.^21^

### Methodology

Data were collected through face-to-face interviews with the household respondents by trained field investigators using the EpiCollect Android application on tablet computers.^22^ The waist circumference of the participants was measured using a non-stretchable tape, to the nearest of 0.1 cm. Body weight was measured on an electronic scale with a capacity of 200 kg and a precision of 50 g. The BP was measured using an electronic sphygmomanometer with an appropriate cuff size covering 80–100% of the circumference of the arm, as per standard guidelines, using an Omron BP monitor (Omron, Kyoto, Japan). Three BP readings at intervals of 5 min were recorded, of which the first reading was discarded and the average of the second and third readings was accepted as the estimated BP reading.^23^

The participants detected with AO were further assessed for TGs, HDL and fasting blood glucose levels. A trained phlebotomist collected fasting venous blood samples of 5 ml aseptically from the selected participants. Samples were transported from the field to the central laboratory for analysis. Fasting plasma glucose was estimated by the glucose oxidase–peroxidase (GOD-POD) method using an Ortho Clinical Diagnostics kit (Ortho Clinical Diagnostics, Raritan, NJ, USA). Serum samples were estimated for TGs and HDL using an automated analyser (Vitros 5600, Ortho Clinical Diagnostics).

### Explanatory variables

Sociodemographic variables considered in this study were age of the respondents (18–39, 40–59, ≥60 y), gender (male, female), level of education (illiterate, primary, secondary, ≥high school), per capita income (≤median: <INR 46 089, >median: >INR 46 095). Lifestyle and clinical factors considered were the presence of tobacco smoking (no, yes) and alcohol consumption (no, yes), dietary habits (vegetarian, non-vegetarian), self-reported DM and HTN (no, yes) and self-reported family history of DM and HTN (no, yes) and type of health facility accessed (government, private, mix). Sedentary lifestyle was assessed from self-reported levels of physical activity, where participants with an absence of regular exercise (brisk walking for at least 5 d and 30 min/d) or involved in occupations not involving vigorously intensive activities were classified as having a sedentary lifestyle, while those involved in regular exercise or in occupations involving vigorously intensive activities were considered as not having a sedentary lifestyle. Body mass index (BMI) was categorized using the Asian cut-off: underweight (BMI <18.5), normal (BMI 18.5–22.9), overweight (BMI 23.0–24.9) and obese (BMI ≥25.0).^24^

### Statistical analysis

Descriptive analysis was undertaken to show the distribution of sociodemographic and lifestyle characteristics of the sample. A binary logistic regression model was applied to assess the relationship of MetS with demographic, socio-economic, biological and behavioural factors. The odds ratio (OR), adjusted odds ratio (aOR) and 95% confidence interval (CI) were reported. Variables for the multivariate analysis were included using backward selection method. A p-value <0.05 was considered statistically significant for the final model. Model diagnostics such as variation inflation factor and goodness of fit were applied for the final adjusted model. Similarly, binary logistic regression was performed to check for associations of non-adherence for DM and HTN medications with adherence among previously diagnosed DM and HTN individuals. All analyses were performed using Stata version 15.1 (StataCorp, College Station, TX, USA).

## Results

We screened a total of 1910 adults for AO, of which 996 (52.15% [95% CI 49.90 to 54.38]), including 298 (37.06% [95% CI 33.79 to 40.46]) males and 698 (63.11% [95% CI 60.22 to 65.91]) females were found to have AO, with the prevalence of AO being significantly higher (χ^2^=126.56, p<0.001) in females than males. Similarly, those 40–59 y of age had a significantly higher prevalence (χ^2^=98.23, p<0.001) of abdominal obesity (67.03% [95% CI 62.97 to 70.86]) as compared with those 18–39 y of age (42.57% [95% CI 39.68 to 45.51]). Female participants had a 1.7 times higher prevalence ratio of having AO compared with males, while the elderly had an about 1.5 times higher prevalence ratio of AO compared with those <40 y of age (Table 1).

**Table 1.** Prevalence of AO according to sex and age group

					Multivariate analysis	
Variable	Examined, n	AO, n	Prevalence, % (95% CI)	Univariate analysis	aOR (95% CI)	p-Value
Total	1910	996	52.15 (49.90 to 54.38)	–	–	
Sex						
Male	804	298	37.06 (33.79 to 40.46)	χ^2^ test; χ^2^= 26.56, **p<0.001**	Ref	**<0.001**
Female	1106	698	63.11 (60.22 to 65.91)		1.72 (1.56 to 1.90)	
Age group (years)						
18–39	1104	470	42.57 (39.68 to 45.51)	χ^2^ test for trends; χ^2^=98.23, **p<0.001**	Ref	**<0.001**
40–59	546	366	67.03 (62.97 to 70.86)		1.59 (1.45 to 1.73)	
≥60	260	260	61.54 (55.47 to 67.27)		1.47 (1.32 to, 1.65)	

Bold P-value represents significant level (P<0.05)

A total of 400 participants were selected randomly from this AO subgroup for further biochemical assessment for MetS. Their sociodemographic characteristics are reported in Table 2. Among these participants screened for MetS (n=400), a majority were female (77%), age <60 y (81%), obese (67.34%), consumed a non-vegetarian diet (69.75%), but did not have a sedentary lifestyle (76%). The prevalence of self-reported DM and HTN was 9.75% and 19.75%, respectively.

**Table 2.** Sociodemographic and lifestyle characteristics of participants screened for MetS (N=400)

Characteristics	Values, n (%)
Sex	
Male	92 (23.00)
Female	308 (77.00)
Age (years)	
18–39	178 (44.50)
40–59	146 (36.50)
≥60	76 (19.00)
Education level	
Illiterate	92 (23.00)
Primary	71 (17.75)
Secondary	59 (14.75)
High school certificate and above	178 (44.50)
Per capita income	
≤Median (<INR 46 089)	361 (90.25)
>Median (>INR 46 095)	39 (9.75)
BMI	
Underweight/normal	52 (13.07)
Overweight	78 (19.60)
Obese	268 (67.34)
Tobacco smoking	
No	363 (90.75)
Yes	37 (9.25)
Alcohol consumption	
No	367 (91.75)
Yes	33 (8.25)
Sedentary lifestyle	
No	304 (76.00)
Yes	96 (24.00)
Dietary habits	
Vegetarian	121 (30.25)
Non-vegetarian or both	279 (69.75)
Previously diagnosed comorbidities	
HTN	79 (19.75)
DM	39 (9.75)
Family history of HTN	
No	353 (88.25)
Yes	47 (11.75)
Family history of DM	
No	363 (90.75)
Yes	37 (9.25)
Health facility accessed	
Government	164 (41.00)
Private	63 (15.75)
Mix	173 (43.25)

On biochemical testing, 135 (33.75%) individuals were detected with elevated TGs and 172 (43%) with low HDL. Furthermore, there were 145 (36.25%) patients with DM and 226 (56.50%) patients with HTN, of which 106 cases of DM and 147 cases of HTN were newly diagnosed, having been detected on screening, while 39 and 79 were previously diagnosed cases of DM and HTN, respectively.

A total of 211 participants with AO had evidence of MetS (52.75% [95% CI 47.83 to 57.62], N=400). Taking the prevalence of AO as 52.15% (996*100/1910) in the source population and the prevalence of MetS among individuals with AO as 52.75% (211/400), it can be estimated that the prevalence of MetS in the source population is 27.51% (52.75%*52.15%). Similarly, the prevalence of MetS in the source population was significantly higher among females (30.73%) as compared with males (24.57%). Further, the prevalence of MetS in the source population increased significantly with age (≥60 y: 43.72%; 40–59 y: 39.02%; 18–39 y: 17.22%). The most prevalent combination of MetS clustering was for all five components (AO, DM, HTN, HDL, TGs; 14.69%), followed by AO, DM and HTN (12.32%) (Table 3).

**Table 3.** Distribution of MetS components by combination (N=211)

Combination	Values, n (%)
AO, DM, HTN	26 (12.32)
AO, DM, HDL	19 (9.00)
AO, DM, TGs	6 (2.84)
AO, HTN, HDL	24 (11.37)
AO, HTN, TGs	19 (9.00)
AO, HDL, TGs	16 (7.58)
AO, DM, HTN, HDL	14 (6.64)
AO, DM, HTN, TGs	23 (10.90)
AO, DM, HDL, TGs	12 (5.69)
AO, HTN, HDL, TGs	21 (9.95)
AO, DM, HTN, HDL, TGs	31 (14.69)

On univariate analysis, the prevalence rate of MetS among individuals with AO was significantly higher in males compared with females (66.30% vs 48.70%, χ^2^=8.81, p=0.003) and older (age ≥60 y) compared with those ≤59 y of age (71.05% vs 58.22%, χ^2^=22.77, p<0.001). However, on adjusted analysis, the odds of having MetS was found to be independently associated with increasing age (≥40 y) but not with gender (Table 4).

**Table 4.** Prevalence of MetS in those with AO according to various attributes (N = 400)

					Multivariate analysis		
Variables	Examined, n	Patients with MetS, n	Prevalence, % (95% CI)	Univariate analysis (χ^2^ test)	Unadjusted OR (95% CI)	aOR^a^ (95% CI)	p-Value
Total	400	211	52.75 (47.83 to 57.62)	–	–	–	–
Sex							
Male	92	61	66.30 (55.99 to 75.27)	χ^2^=8.81, **p=0.003**	Ref	Ref	0.108
Female	308	150	48.70 (43.13 to 54.30)		0.48 (0.30 to 0.78)	0.62 (0.35 to 1.11)	
Age (years)							
18–39	178	72	40.45 (33.45 to 47.86)	χ^2^=22.77, **p<0.001**	Ref	Ref	**<0.001**
40–59	146	85	58.22 (50.02 to 65.99)		2.05 (1.32 to 3.20)	1.94 (1.23 to 3.07)	
≥60	76	54	71.05 (59.81 to 80.19)		3.61 (2.03 to 6.45)	3.38 (1.87 to 6.12)	
Education level							
Illiterate	92	52	56.52 (46.17 to 66.33)	χ^2^=4.82, p=0.18	Ref	–	–
Primary	71	44	61.97 (50.11 to 72.56)		1.25 (0.67 to 2.36)		
Secondary	59	30	50.85 (38.17 to 63.42)		0.80 (0.41 to 1.53)		
≥High school certificate	178	85	47.75 (40.47 to 55.13)		0.70 (0.42 to 1.17)		
Per capita income							
≤Median (<INR 46 089)	361	193	53.46 (48.28 to 58.58)	χ^2^=0.75, p=0.38	Ref	–	–
>Median (>INR 46 095)	39	18	46.15 (31.13 to 61.91)		0.75 (0.38 to 1.45)		
BMI							
Underweight/normal	52	26	50.00 (36.57 to 63.43)	χ^2^=0.41, p=0.81	Ref	–	–
Overweight	78	40	51.28 (40.20 to 62.23)		1.05 (0.52 to 2.12)		
Obese	268	145	54.10 (48.08 to 60.01)		1.18 (0.65 to 2.14)		
Tobacco smoking							
No	363	185	50.96 (45.81 to 56.10)	χ^2^=5.02, **p=0.025**	Ref	Ref	0.535
Yes	37	26	70.27 (53.58 to 82.88)		2.27 (1.09 to 4.74)	1.32 (0.55 to 3.16)	
Sedentary lifestyle							
No	304	159	52.30 (46.66 to 57.89)	χ^2^=0.10, p=0.75	Ref	–	–
Yes	96	52	54.17 (44.08 to 63.92)		1.08 (0.68 to 1.71)		
Dietary habits							
Vegetarian	121	63	52.07 (43.13 to 60.87)	χ^2^=0.03, p=0.856	Ref	–	–
Non-vegetarian or both	279	148	53.05 (47.15 to 58.86)		1.04 (0.68 to 1.59)		
Family history of HTN							
No	353	184	52.12 (46.89 to 57.32)	χ^2^=0.47, p=0.49	Ref	–	–
Yes	47	27	57.45 (42.89 to 70.81)		1.24 (0.67 to 2.29)		
Family history of DM							
No	363	188	51.79 (46.63 to 56.92)	χ^2^=1.45, p=0.229	Ref	–	–
Yes	37	23	62.16 (45.53 to 76.35)		1.53 (0.76 to 3.07)		
Health facility accessed							
Government	164	77	46.95 (39.40 to 54.65)	χ^2^=7.12, **p=0.028**	Ref	Ref	0.1047
Private	63	42	66.67 (54.08 to 77.26)		2.26 (1.23 to 4.15)	1.98 (1.05 to 3.73)	
Mix	173	92	53.18 (45.68 to 60.53)		1.28 (0.84 to 1.97)	1.22 (0.78 to 1.91)	

a p<0.05 is considered statistically significant.

Bold P-value represents significant level (P<0.05).

Model goodness of fit, p=0.4964.

Among the 34 previously diagnosed cases of DM, a total of 28 (82.35%) were adherent to their anti-diabetes medication, while in the 51 previously diagnosed cases of HTN, 28 (54.90%) were adherent to their anti-hypertensive medication. Medications for dyslipidaemia were taken by only 17 individuals among those detected with MetS. Among individuals with DM, females (84.00%), those 40–59 y of age (90.00%) and underweight/normal individuals (83.33%) were more likely to be adherent to their anti-diabetes medications, while in patients with HTN, those ≥60 y of age (63.64%), overweight (58.33%) and accessing private health facilities (66.67%) were more likely to be adherent to their anti-hypertensive medications, although none of the associations was statistically significant (Table 5).

**Table 5.** Medication adherence pattern among previously diagnosed DM and HTN patients on treatment

	Previously diagnosed with DM on treatment (N=34)			Previously diagnosed with HTN on treatment (N=51)		
Variables	Non-adherent (n=6), n (%^a^)	Adherent (n=28), n (%^a^)	Unadjusted OR (95% CI), p-value	Non-adherent (n=23), n (%^a^)	Adherent (n=28), n (%^a^)	Unadjusted OR (95% CI), p-value
Sex			p=0.676			p=0.843
Male	2 (22.22)	7 (77.78)	Ref	6 (42.86)	8 (57.14)	Ref
Female	4 (16.00)	21 (84.00)	1.50 (0.22 to 10.04)	17 (45.95)	20 (54.05)	0.88 (0.26 to 3.05)
Age (years)			p=0.46			p=0.5047
18–39	0 (0.0)	0 (0.0)	–	3 (60.00)	2 (40.00)	Ref
40–59	1 (10.00)	9 (90.00)	Ref	12 (50.00)	12 (50.00)	1.50 (0.21 to 10.65)
≥60	5 (20.83)	19 (79.17)	0.42 (0.04 to 4.16)	8 (36.36)	14 (63.64)	2.63 (0.36 to 19.18)
Education level			p=0.91			p=0.5067
Illiterate	2 (16.67)	10 (83.33)	Ref	4 (36.36)	7 (63.64)	Ref
Primary	1 (16.67)	5 (83.33)	1.00 (0.07 to 13.87)	6 (46.15)	7 (53.85)	0.67 (0.13 to 3.45)
Secondary	0 (0.00)	3 (100.00)	–	6 (66.67)	3 (33.33)	0.29 (0.04 to 1.82)
≥High school certificate	3 (23.08)	10 (76.92)	0.67 (0.09 to 4.89)	7 (38.89)	11 (61.11)	0.90 (0.19 to 4.23)
Per capita income						p=0.487
≤Median (<INR 46 089)	6 (19.35)	25 (80.65)	–	20 (43.48)	26 (56.52)	Ref
>Median (>INR 46 095)	0 (0.00)	3 (100.00)	–	3 (60.00)	2 (40.00)	0.51 (0.08 to 3.37)
BMI			p=0.99			p=0.951
Underweight/normal	1 (16.67)	5 (83.33)	Ref	2 (50.00)	2 (50.00)	Ref
Overweight	2 (18.18)	9 (81.82)	0.90 (0.06 to 12.58)	5 (41.67)	7 (58.33)	1.40 (0.14 to 13.57)
Obese	3 (17.65)	14 (82.35)	0.93 (0.08 to 11.18)	16 (45.71)	19 (54.29)	1.19 (0.15 to 9.41)
Tobacco smoking			p=0.1938			p=0.1294
No	4 (13.79)	25 (86.21)	Ref	18 (40.91)	26 (59.09)	Ref
Yes	2 (40.00)	3 (60.00)	0.24 (0.03 to 1.92)	5 (71.43)	2 (28.57)	0.28 (0.05 to 1.59)
Sedentary lifestyle			p=0.537			p=0.5092
No	4 (15.38)	22 (84.62)	Ref	16 (48.48)	17 (51.52)	Ref
Yes	2 (25.00)	6 (75.00)	0.55 (0.08 to 3.73)	7 (38.89)	11 (61.11)	1.48 (0.46 to 4.76)
Dietary habits			p=0.255			p=0.082
Vegetarian	1 (7.69)	12 (92.31)	Ref	4 (26.67)	11 (73.33)	Ref
Non-vegetarian or both	5 (23.81)	16 (76.19)	0.27 (0.03 to 2.59)	19 (52.78)	17 (47.22)	0.33 (0.09 to 1.22)
Family history of DM						p=0.810
No	6 (20.00)	24 (80.00)	–	21 (45.65)	25 (54.35)	Ref
Yes	0 (0.00)	4 (100.00)	–	2 (40.00)	3 (60.00)	1.26 (0.19 to 8.26)
Family history of HTN						p=0.110
No	6 (18.75)	26 (81.25)	–	22 (50.00)	22 (50.00)	Ref
Yes	0 (0.00)	2 (100.00)	–	1 (14.29)	6 (85.71)	6.00 (0.67 to 54.04)
Health facility accessed			p=0.830			p=0.4686
Government	3 (21.43)	11 (78.57)	Ref	8 (40.00)	12 (60.00)	Ref
Private	0 (0.00)	8 (100.0)	–	3 (33.33)	6 (66.67)	1.33 (0.26 to 6.94)
Mix	3 (25.00)	9 (75.00)	0.82 (0.13 to 5.08)	12 (54.55)	10 (45.45)	0.56 (0.16 to 1.89)

a Row-wise percentages given.

## Discussion

The present study found that nearly half of adult individuals in an urban slum and slum resettlement colony in Delhi had AO on screening, while more than half of the individuals with AO were detected as having MetS on biochemical evaluation. Previous studies from urban slums in Delhi and Hyderabad have also reported more than one in four adult individuals with MetS, suggestive that the overall prevalence of the condition has been stable in the past 2 decades in these low-resource settings.^6,7^

In this study, the prevalence rate of MetS was found to be significantly higher in men (66.30%) compared with women (48.70%), based on the initial univariate analysis. This finding, while in agreement with the evidence from some previous studies,^9,10^ also contradicts some other studies.^13,14^ However, this significance did not hold in the subsequent multivariable analysis, suggestive of the observed difference being attributable to confounding variables. Also, in this study, women had a higher prevalence of AO compared with men.

Furthermore, the prevalence of MetS was observed to be higher in older and elderly adults, a finding consistent with prior evidence that is likely due to the increasing risk of DM, HTN and obesity associated with aging.^1,15^ As the prevalence of DM, HTN and dyslipidaemia was higher in males, this may translate into an increased risk for MetS. Furthermore, in this study alcohol use was reported only by men, which is a known risk factor associated with MetS.^25^

MetS represents an NCD multimorbid condition in which poor adherence to medications resulting in uncontrolled blood sugar, BP and elevated TG levels contributes to worsening of patient health outcomes and increased risk of cardiovascular disease complications.^26^ In this study, medication non-adherence, a major public health challenge among patients with NCDs in India, as in other resource-limited settings, was observed in nearly half of the previously diagnosed patients with HTN and one in five previously diagnosed cases of DM.^27^ Furthermore, our study findings indicate that only a small proportion of the patients with elevated TGs in individuals with MetS were on lipid-lowering therapy, which increased their risk of progression of cardiovascular disease.^28^ Previous studies in low- and middle-income countries and also China have reported a similar phenomenon of reduced awareness of dyslipidaemia in patients contributing to ineffectual management of this high-risk condition.^29,30^

### Study implications

India's national program for NCDs mandates free of cost opportunistic and community-based screening of DM, HTN and AO,^31^ but it may be insufficient for detection of metabolic syndrome (MetS) in socio-economically disadvantaged Indian populations residing in urban slums. A significant proportion of the population at risk of MetS in India is likely to have an additional dyslipidaemia component that is not routinely screened for within the public health system. Furthermore, evidence from large surveys estimates that nearly 40% of Indian patients with DM and HTN remain undiagnosed, indicative of health system inefficiency.^5^

Strengthening the public health system to facilitate early detection of MetS and ensuring its standard care would require diagnostic and therapeutic investments through leveraging existing primary care infrastructure and personnel in health and wellness centres and primary health facilities. Further, failure to adequately treat MetS represents a missed opportunity to prevent or delay the progression of cardiovascular disease. Although the cost-effectiveness of cholesterol screening and treatment is well-established in developed countries, it may require additional economic evaluation in resource-limited settings.^32^ Post-screening activities apart from therapeutic interventions also require a focus on evidence-based health promotion activities to improve diet and physical activity that are tailored and beneficial in resource-limited settings such as urban slums with prevalent adverse social determinants. Consequently, the present study has a programmatic recommendation for universal screening of adult individuals with AO for DM, HTN and TGs at regular intervals, with recommendations for suitable lifestyle modifications involving diet and exercise.

The present study has certain strengths. The prevalence of risk factors for cardiac markers was assessed along with clinical and biochemical features of MetS. A robust sampling protocol was used to assess the risk factors of MetS in the target population. However, there are also some potential limitations of this study. First, this was a cross-sectional study and could not establish causality between MetS and its predictors. Second, it was a single-site study with a limited sample size that may preclude extrapolation of the findings to other settings. Third, there is a possibility of reporting bias for self-reported data such as family history, medications, etc. Medication adherence may be overestimated due to social desirability bias.

## Conclusions

In conclusion, a high burden of MetS and suboptimal treatment status is prevalent in urban slum populations. Screening of individuals with AO, especially those >40 y of age, in such settings can be an effective programmatic strategy for early diagnosis and management of MetS and its underlying components. Sensitization of healthcare providers in low-resource settings for the promotion of medication adherence in cases of DM and HTN, with treatment initiation for clinical management of cases of dyslipidaemia towards control of MetS, warrant high prioritization.

## Data Availability

The data are available upon request to the corresponding author.
